# Late onset periprosthetic infection of the hip caused by the fish pathogen *Lactococcus garvieae* in a patient not associated with fish exposure

**DOI:** 10.7150/jbji.43655

**Published:** 2020-04-06

**Authors:** Marianne Westberg, Hanne Brekke, Nils Olav Hermansen, Bernhard Flatøy

**Affiliations:** 1 Division of Orthopaedic Surgery, Oslo University Hospital, Oslo, Norway; 2 Department of Medical Microbiology, Oslo University Hospital, Oslo, Norway

**Keywords:** * Lactococcus garvieae*, fish pathogen, periprosthetic hip infection, two-stage surgery

## Abstract

*Lactococcus garvieae* is a fish pathogen, rarely causing opportunistic infections in humans. There are only a few cases reported in the literature, mainly endocarditis, suggesting an association with raw fish consumption. We report a case of a periprosthetic hip infection successfully treated with a two-stage revision surgery.

## Introduction

*Lactococcus garvieae* (*L. garvieae*) is traditionally known as a fish pathogen causing septicemia in fish species like: rainbow trout, gray mullet and giant freshwater prawns 1-3. It can also cause mastitis in ruminants, and has been isolated from dairy products and meat 4-6. *L. garvieae* infection in humans have been associated with aquaculture practice 3,4, and with ingestion of contaminated fish 6. In 1995 Facklam and colleagues suggested that *Lactococci* were probably opportunistic pathogens mainly affecting persons with underlying debilitations and diseases 7. In 2016 Yooseok and colleagues reviewed the reported human cases thus far of *L. garvieae* associated infections, and found that in 19 reported cases nine (47, 4%) were associated with fish-exposure and 12 (63,2%) of the affected patients had a known gastrointestinal disorder 8. The 1985 *Bergey's Manual of Systematic Bacteriology* split the *Streptococcu*s genus into three genera: *Streptococcus*, *Enterococcus* and *Lactococcus.* The *Lactococcus* genus are facultative anaerobic, catalase-negative, and gram-positive cocci, which occur singly, in pairs, or in chains. Several species and subspecies have been described. These bacteria are physiologically similar to the serogroup D fecal streptococci and resemble enterococci 7. There have been few reports of human *L. garvieae* infections in the literature, but in recent years an increasing number of cases have been reported, the majority being endocarditis 9. There has only been one previous case report of periprosthetic hip infection due to *L. garvieae*, this was in a fishmonger 10. We present the first published case of *L. garvieae* infection in Norway; a periprosthetic late onset hip infection in a patient who denies eating or handling fish.

## Case Report

In 2016, an 80-year old man was admitted to our emergency department due to acute onset of right hip and groin pain for four days. His past medical history included chronic obstructive pulmonary disease (COPD), emphysema grade 3 according to the GOLD classification, as well as hypertension, and hypercholesterolemia. Eight months earlier he was diagnosed with a locally invasive prostate cancer, Gleason score 8, treated with radiotherapy and adjuvant hormone treatment. A right hip pertrochanteric fracture had been treated with an osteosynthesis and re-operated due to avascular necrosis 15 and 13 years earlier, respectively. Infection was suspected at the time of revision. The osteosynthesis was hence removed, a resection of the femoral head was performed, and a cement-spacer placed. There was no growth in the perioperatively obtained biopsies, and a cemented total hip arthroplasty (THA) (Spectron^TM^, Smith & Nephew) was then performed a few weeks later. The THA had been functioning well over the past 13 years.

On clinical examination he was afebrile, though infection of the THA was suspected. Laboratory tests revealed C-reactive protein (CRP) of 300 mg/L and leukocyte count of 9.0 x 10^9^/L. Two sets of blood cultures were negative after seven days of incubation, according to endocarditis guidelines with prolonged incubation. An arthrocentesis of the hip revealed purulent synovial fluid. Microscopy of the synovial fluid showed increased amount of polymorphonuclear leukocytes, supporting the suspicion of an ongoing infectious process. The alfa-defensin test was positive. Radiographic imaging of the right hip revealed signs of loosening of both the stem and the acetabular component, as well as wear of the polyethylene liner. A two-stage exchange arthroplasty was planned. He was operated 6 days after admission with removal of the prosthesis and cement as well as a thorough soft tissue revision. A gentamicin-loaded spacer was placed. Empiric intravenous antimicrobial therapy with vancomycin 1 g times 2 and cloxacillin 2 g times 4 per day was started.

The biopsies were cultured aerobically and anaerobically on the following in house produced media: Chocolate blood agar, sheep blood agar, lactose agar, mannitol salt agar, anaerobic blood agar, anaerobic blood agar with kanamycin, and anaerobic blood agar with kanamycin and vancomycin. The media were incubation in 35˚ Celcius, CO2 enriched atmosphere for seven days, according to Norwegian guidelines for culturing biopsies. A semiquantitative measurement method revealed moderate growth of alfa hemolytic grey-white colonies on chocolate and sheep blood agar in six of six biopsy-samples on day one of incubation. On microscopic examination of the growth gram positive short chains of cocci were identified. The microbe was identified as *Lactococcus garvieae* on matrix-assisted laser desorption/ ionization time of flight mass spectrometry (MALDI-TOF Bruker) with a score of 2,09, with MALDI Biotyper MSP Identification Standard Method 1.1. The identification was further verified by 16s rDNA PCR, where 784 of 784 (100%) base pairs were sequenced without any mismatch, and an NCBI blast identified the microbe as *L. garvieae*.

Upon identification of the causative microbe, antimicrobial treatment was changed to aminopenicillin according to the pattern of antibiotic susceptibility (Table 1). Antimicrobial susceptibility testing was performed with a quantitative assay for determining the Minimum Inhibitory Concentration (MIC) of antimicrobial agents with Liofilchem MIC Test Strips. Known EUCAST breakpoints were not available for the causative agent. On the fifth post-operative day the patient developed acute on chronic renal failure, with an increase in serum creatinine from 130 µmol/L on admission to 625 µmol/L, a hemodialysis was commenced. It was believed that the development of acute on chronic renal failure was caused by consumption of nephrotoxic medications including vancomycin, NSAIDs and angiotensin 2 blockers, and these medications were stopped. On the 7^th^ postoperative day the haemoglobin level dropped from 9,2 to 5,9 g/dL, and he received a blood transfusion. Gastroscopy revealed two ulcers, one in the duodenum and one in the antrum. Due to the renal failure the dose of aminopenicillin was reduced to 1 g times 2 daily.

The patient gradually improved, and was eventually no longer in need of hemodialysis. After 18 days of intravenous antimicrobial treatment, he was transferred to rehabilitation and the treatment was changed to oral aminopenicillin for another 5,5 weeks. The second stage of surgery was performed after a 5-week antibiotic free period. At the time of re-implantation, there were no clinical signs of local infection, and the CRP was normal. He was re-implanted with an uncemented Corail revision stem (Depuy International) and an uncemented trabecular metal cup (ZimmerBiomet). Except for antimicrobial prophylaxis given perioperatively, no additional antimicrobial therapy was given. Six tissue samples were taken during re-implantation, all of which were culture negative after seven days of culturing. The recovery was slow and uneventful, with a favourable outcome at 2 years follow-up. There were no clinical signs of infection, radiographic- imaging showed no signs of loosening of the prosthesis, and CRP and leukocytes were normal. The patient walked without aid, only restricted by his underlying COPD.

## Discussion

*L. garvieae* is known to be responsible for septicaemia in various fish species and aquaculture practice. Endemic infections in fish usually occur in the summer, and are suggested to correlate with outbreaks of *L. garvieae* infections in humans as well. It rarely affects humans, though, and is infrequently found in clinical routine samples. There are only a few reports of* L. garvieae* related infections in humans in the literature, but the number of reported cases has increased in the last decades. The majority of reported cases are due to bacteremia and endocarditis. Affected patients commonly have multiple comorbidities, especially heart disease and gastrointestinal disorders. To our knowledge there has only been one previous case report of periprosthetic hip infection and one case of knee prosthesis infection due to* L. garvieae* 10,11*.* Manipulation and consumption of raw fish have by most authors been thought to be the most likely sources of *L. garviaea* infections in humans. In both the previous reports of periprosthetic infections, this was the case.

Our patient was an elderly man with a severe COPD and an underlying malignant disease, but with no known heart or gastrointestinal tract diseases prior to admission. He had no recent surgical or dental procedures, or known skin defects. Further he denied any association with handling or consuming raw fish or unpasteurized milk. The periprosthetic hip-infection in this patient occurred during the winter-months. We could not identify the source of infection, neither did the patient have many similarities with previous reported cases of *L. garviae* infection. He did though develop gastrointestinal bleeding during treatment, which could indicate an underlying occult gastrointestinal ulcer-disease.

Aminopenicillins and cephalosporins are generally effective in the treatment of *L. garvieae* 12. A two-stage procedure is regarded as the gold standard in treating periprosthetic infections, and traditionally the success rate of a two-stage procedure is reported to be 80-100% depending on host factors and bacterial species. The patient responded well to antimicrobial therapy, without signs of relapse at 2-year follow-up.

## Conclusion

Although rarely isolated in human infections, *L. garvieae* may cause severe disease including periprosthetic infections. Despite repeated specific questioning we could not identify the source of the microbe. As the numbers of immunocompromised elderly patients with severe comorbidities are increasing, periprosthetic infections with uncommon low virulence bacteria, like *L. garvieae,* are more likely to be found, and clinicians and microbiologist should be aware of this. In the presented case, a standard two-stage surgery with a 7-week course of antimicrobial treatment eradicated the infection successfully.

## Figures and Tables

**Table 1:** Antimicrobial-susceptibility with determination of MIC for *Lactococcus garvieae* in the reported case.

Antibiotic	MIC (mcg/ml)
Ampicillin	0.25
Ciprofloxacin	1.0
Clindamycin	>256
Ceftriaxon	0.5
Cefotaxime	0.5
Gentamicin	4
Meropenem	0.064
Penicillin	0.5
Rifampin	>32
Trimetoprim sulpha	1
Vancomycin	0.5

MIC was determined with Liofilchen MIC Test strips. (EUCAST breakpoints were not available for *L. garvieae*.)
